# Nasopharyngeal Epstein-Barr Virus Load: An Efficient Supplementary Method for Population-Based Nasopharyngeal Carcinoma Screening

**DOI:** 10.1371/journal.pone.0132669

**Published:** 2015-07-07

**Authors:** Yufeng Chen, Weilin Zhao, Longde Lin, Xue Xiao, Xiaoying Zhou, Huixin Ming, Tingting Huang, Jian Liao, Yancheng Li, Xiaoyun Zeng, Guangwu Huang, Weimin Ye, Zhe Zhang

**Affiliations:** 1 Department of Otolaryngology-Head & Neck Surgery, First Affiliated Hospital of Guangxi Medical University, Nanning, Guangxi, China; 2 Key Laboratory of High-Incidence-Tumor Prevention & Treatment (Guangxi Medical University), Ministry of Education, Nanning, Guangxi, China; 3 Department of Epidemiology, School of public health, Guangxi Medical University, Nanning, Guangxi, China; 4 Cancer Institute of Cangwu County, Wuzhou, Guangxi, China; 5 Department of Medical Epidemiology and Biostatistics, Karolinska Institutet, Stockholm, Sweden; 6 Department of Environmental and Molecular Medicine, Mie University Graduate School of Medicine, Mie, Japan; Gustave Roussy, FRANCE

## Abstract

Serological detection of Epstein-Barr virus (EBV) antibodies is frequently used in nasopharyngeal carcinoma (NPC) mass screening. However, the large number of seropositive subjects who require close follow-up is still a big burden. The present study aimed to detect the nasopharyngeal EBV load in a high-risk population seropositive for antibodies against EBV, as well as to examine whether assay for nasopharyngeal EBV DNA load might reduce the number of high-risk subjects for follow-up and improve early detection of NPC. A prospective and population-based cohort study was conducted in southern China from 2006 through 2013. Among 22,186 participants, 1045 subjects with serum immunoglobulin A (IgA) antibodies against viral capsid antigen (VCA) titers ≥ 1:5 were defined as high-risk group, and were then followed-up for NPC occurrence. Qualified nasopharyngeal swab specimens were available from 905 participants and used for quantitative PCR assay. Our study revealed that 89% (802/905) subjects showed positive EBV DNA in nasopharyngeal swab. The nasopharyngeal EBV load in females was higher than that in males. The nasopharyngeal EBV load increased with increasing serum VCA/IgA titers. Eight cases of newly diagnosed NPC showed an extremely elevated EBV load, and 87.5% (7 of 8 patients) were early-stage NPCs. The EBV loads of 8 NPCs were significantly higher than those of 897 NPC-free subjects (mean, 2.8×10^6^ copies/swab [range 4.8×10^4^-1.1×10^8^] vs. 5.6×10^3^ [range 0-3.8×10^6^]). Using mean EBV load in NPC-free population plus two standard deviations as cut-off value, a higher diagnostic performance was obtained for EBV load test than serum VCA/IgA test (area under ROC, 0.980 vs 0.895). In conclusion, in a prospective and population-based study we demonstrated that an additional assay of EBV load in the nasopharynx among high-risk individuals may reduce the number of subjects needed to be closely followed up and could serve as part of a NPC screening program in high-risk populations.

## Introduction

Nasopharyngeal carcinoma (NPC) is one of the most common head and neck cancers in Southern China. The close association of NPC with Epstein-Barr virus (EBV) has been demonstrated by genetic analysis as well as by serological studies [1]. In endemic regions, almost 100% of undifferentiated-type NPC tumor cells carry EBV genome and express EBV proteins [2]. Seroepidemiologic studies have proved that NPC patients have significantly higher levels of antibodies against EBV antigens, e.g. IgA antibodies against viral capsid antigen (VCA/IgA) and early antigen (EA/IgA). EBV VCA/IgA and EA/IgA antibodies measured by immunofluorescent or Immunoenzymatic assays have been used for the serologic screening of NPC in Wuzhou, China [3]. Other population-based prospective studies conducted in Guangdong province and Taiwan confirmed the feasibility of using EBV antibodies as a screening tool for NPC and revealed that individuals with elevated EBV antibody levels have a significantly increased risk of NPC development [4–6]. Furthermore, elevation of the EBV antibody levels precedes the clinical onset of NPC within a window of 37 months [5]. In recent years, enzyme-linked immunosorbent assays (ELISA) using recombinant or synthetic EBV antigens are increasingly advocated to replace the traditional immunofluorescent or Immunoenzymatic assays [7–9]. EBV-IgA assay using finger-prick dried blood samples has also been proposed for field screening to identify "at-risk" persons because of the easiness of sample collection, storage, and transportation [10]. However, to date, these serologic screening tests have not reached satisfactory levels to improve the accuracy of diagnosing NPC and to predict NPC development in seropositive high-risk individuals.

Cell-free EBV DNA can be found in the plasma and serum of NPC patients. In addition to serological assays, quantitative evaluation of EBV DNA load in the circulation has also been shown to be a sensitive molecular tool for detecting and monitoring tumor recurrence of NPC [11]. But the potential value for screening to identify early stage NPC is limited. This might be due to the fact that early stage NPC releases only a limited amount of viral DNA to the blood, which is undetectable in the circulation [12]. NPC primarily arises from the epithelial cells in the Rosenmüller’s fossa or the post-wall of the nasopharyngeal cavity. Elevated EBV-associated antibodies in NPC patients are mostly IgA class antibodies of mucosal origin. EBV genome can be detected in neoplastic cells of virtually all NPC cases [2]. Clonal EBV genome can be consistently detected in invasive carcinomas and pre-cancerous high-grade dysplastic lesions [13]. These clues suggest that detecting EBV genome in specimens collected directly from the nasopharyngeal region via brushing or swabbing should be highly predictive for the screening of asymptomatic NPC. Several groups have reported promising results by showing significantly higher swab/brush EBV DNA load in NPC patients [14, 15]. However, so far there is no prospective study to address the feasibility of screening NPC by evaluating EBV DNA load in the nasopharynx.

In this cohort study, for the first time, we addressed the presence of EBV DNA in the nasopharynx of a high-risk population of NPC. We analyzed the correlation of nasopharyngeal EBV DNA load and level of serum antibodies against EBV. We also evaluated the diagnostic value of nasopharyngeal EBV DNA load.

## Materials and Methods

### Study populations

Between 2006 and 2013 a population-based NPC screening program was conducted in Cangwu county, Wuzhou, Guangxi Autonomous Region in Southern China. Three towns of Cangwu county were selected to participate in the screening program. All the eligible subjects were asked to participate in the screening tests. Inclusion criteria were: 1) age between 30–59 years; 2) being Cantonese-speaking; 3) without prevalent NPC; 4) having a good physical or psychological condition and consciousness. Those who had severe cardiovascular, liver, or kidney diseases were excluded. A serum sample was taken from each subject at enrollment for detection of EBV VCA/IgA antibody by Immunoenzymatic assay, and each subject was offered an otorhinolaryngologic and neck lymphatic examination. A total of 22186 individuals volunteered to take part in the initial screening program, and the participation rate was approximately 56.2%.

Our study group has strictly abided by the principles of Helsinki Declaration. This study was approved by the Ethics Review Committee of Guangxi Medical University. All the samples were taken with written informed consent from donors.

### Nasopharyngeal swab and blood sample collection

Nasopharyngeal cells were obtained by using a homemade nasopharyngeal swab with outer catheter as described by Shinn-yn Lin *et al*. with slight modification [16]. In brief, the subject's nasal cavity was sprayed with a 1% dicaine solution for superficial anesthesia. The swab with outer catheter was inserted into the nasal cavity and advanced toward the nasopharyngeal wall along the inferior nasal meatus. After the tip of the catheter touched the posterior nasopharyngeal wall, the catheter was withdrawn 2cm while the cotton swab was firmly protruded against the posterior nasopharyngeal wall and swept over the surface of posterior and lateral nasopharyngeal walls 2–3 times. Then the swab was withdrawn until the cotton tip of the swab was completely inside the lumen of the catheter. The cotton stick together with the catheter was removed from the nasopharynx and the nasal cavity. After collecting samples from both left and right sides by using the same swab, the swab was withdrawn from the catheter, and the cotton tip was cut and dipped into 2ml of saline and stored at -80°C before DNA extraction. The venous blood was collected into coagulation-promoting vacuum tubes and centrifuged. Serum was aliquoted into 2ml microtubes and stored at -20°C before use.

### EBV serology

Serum EBV VCA/IgA antibody levels were determined by titration using an Immunoenzymatic assay described previously [17]. In brief, cell smears were prepared from B95-8 cultures, fixed in acetone and used in the indirect Immunoenzymatic method with peroxidase-conjugated anti-human IgA antibody. Sera diluted to 1:5 were added to separate wells of slide. The slides were incubated at 37°C for 30 min in a humid atmosphere, and washed 3 times with phosphate-buffered saline (PBS). Peroxidase-conjugated antihuman IgA antibody in appropriate dilution was added to the slides. The slides were incubated again for 30 min, washed 3 times with PBS, and flooded with diaminobenzidine and H_2_O_2_ for 10 min. Positive and negative control sera were incubated in each experiment. A serum was considered positive if the cells in the well that contained the 1:5 dilution showed brown color characteristic of this test. The blood specimens from persons antibody-positive in the initial screening were tested in further dilutions. The highest dilution of serum still positive for IgA antibody to VCA was considered as the antibody titer of that serum.

### EBV DNA load measurement

DNA from nasopharyngeal swabs was extracted with a QIAamp DNA Mini Kit (Qiagen, Germany) using a protocol recommended by the manufacturer. A final elution volume of 50ul was used. Concentration of total DNA was measured by NanoDrop 2000 (Thermo Scientific, America). Two real-time quantitative polymerase chain reaction (qPCR) systems described previously [18, 19] were set up to detect EBV DNA and the *β-globin* gene. The *β-globin* gene was used as a quality control for the nasopharyngeal swab sampling, DNA extraction and PCR reaction. A standard curve of the CT values obtained from plasmid DNA containing *BamHI-W* or *β-globin* fragment respectively was established in parallel. Each sample was tested in duplicate, and the mean of the two values was taken as the copy number of the sample. Samples were defined as negative if the CT values exceeded 40 cycles. In all experiments appropriate negative and positive controls were included during nucleic acid isolation and amplification. Swab DNA samples were renumbered before EBV DNA load detection to ensure a blind test. The copy numbers of EBV DNA or *β-globin* gene per swab (expressed in copies/swab) were calculated according to the following equation:

$$C=Q\times \frac{V_{DNA}}{V_{PCR}}\times \frac{1}{V_{EXT}}$$

C: target concentration in one swab (copies/swab)

Q: target quantity (copies) determined by PCR

V_DNA_: total volume of DNA obtained after extraction (50ul)

V_PCR_: volume of DNA solution used for PCR (1ul)

V_EXT_: volume of saline solution extracted (1 swab)

### Statistical analysis

All the statistical analyses were performed using the SPSS statistical analysis software (Version 16.0, SPSS Inc., Chicago, IL). EBV DNA load and serum VCA/IgA antibody titers were log-transformed (log10 transformed) to ensure normal distribution of the variables. Normality test of selected variables was performed by the Kolmogorov—Smirnov test. Spearman’s correlation coefficient was calculated for evaluating the correlation between nasopharyngeal EBV DNA load, DNA amount in swabs, serum anti-EBV VCA/IgA titers and age. Comparison of concordance between EBV DNA and serum anti-EBV VCA/IgA results was conducted by calculating prevalence-adjusted bias-adjusted Kappa (PABAK) coefficient. Mann-Whitney U test was performed to compare the differences of EBV DNA load and VCA/IgA antibodies by sex and disease status (NPC vs NPC-free). To evaluate independent effects of various covariates on EBV DNA load, a multivariate linear regression model was established, with log-transformed EBV DNA load as dependent variable. Receiver operating characteristic curve (ROC curve) was also created to evaluate the overall diagnostic values of EBV DNA load and VCA/IgA antibodies. All the statistical tests were two-sided, and a *P* < 0.05 was considered as statistically significant.

## Results

### Screening results

Among the tested subjects, 21116 subjects (95.2%) were VCA/IgA negative, no NPC cases were observed among the seronegative subjects at the first screening and in the follow-up period of 2010–2013. From all the participants, 1070 subjects (4.8%) with VCA/IgA titer ≥ 1:5 were defined as high-risk group. For all the 1070 subjects in the high-risk group, endoscopy and pathological biopsy were performed by otorhinolaryngologists to diagnose NPC. Of them 25 NPC cases were newly found, and the remaining 1045 subjects constituted our study cohort. In 2010, a follow-up serological and endoscopy examination were performed among the 1045 high-risk subjects. Besides serological test, otorhinolaryngologic and neck lymphatic examination, these subjects were asked for donating a nasopharyngeal swab and undergoing a face-to-face interview using a questionnaire. In total, 917 subjects agreed to provide a nasopharyngeal swab. In 2011, these subjects were actively followed up by a repeated examination, and in 2012–2013, were followed up for NPC occurrence by linkage to Cangwu Cancer Registry. Five NPCs were newly diagnosed in year 2010, and 3 in year 2011 (Table 1). No new NPC cases were diagnosed in 2012 and 2013. According to the American Joint Committee on Cancer (AJCC) TNM Staging System (7th ed., 2010), 7 of the 8 cases were early-stage NPCs (2 cases of stageⅠ, 5 cases of stage Ⅱ, and 1 case of stage Ⅲ), and the early diagnosis rate was 87.5%. The results of serological test and EBV viral load from the nasopharyngeal swabs in 2010 were used for analysis in this study.

**Table 1:** Results of a nasopharyngeal carcinoma screening program performed in Cangwu, a high-risk area in China.

	VCA/IgA status^a^		
	Positive	Negative	Total
	No.(%)	No.(%)	No.
**Number of subjects enrolled (2006–2010)**	1070 (4.8%)	21116 (95.2%)	22186
NPC cases detected at enrollment	25 (100%)	0	25
**Follow-up for high-risk subjects**			
1^st^-round follow-up retest VCA/IgA^b^ (2010)	978 (93.6%)	67 (6.4%)	1045
NPC cases detected at 1^st^-round follow-up retest	5 (100%)	0	5
2^nd^-round follow-up retest VCA/IgA^c^ (2011)	896 (97.3%)	25 (2.7%)	921
NPC cases detected until the end of follow-up (2011–13)	3 (100%)	0	3
**NPC cases detected from seronegative subjects (2010–13)**	0	0	0

VCA, viral capsid antigen; NPC, nasopharyngeal carcinoma.

^a^ Serum EBV VCA/IgA titer ≥1:5 designated as seropositive, <1:5 designated as seronegative

^b^ 67 subjects were seronegative and 5 NPC cases were newly diagnosed at the 1^st^-round follow-up retest; these 72 subjects were excluded in the next-round follow-up

^c^ At the 2^nd^-round retest for VCA/IgA, 52 subjects were lost for follow-up.

### EBV DNA load of nasopharyngeal swabs and serum VCA/IgA titer in the seropositive high risk population

To assess the quality of swab sampling, the copies of *β-globin* gene were determined in all 917 swab DNA samples by quantitative PCR. *β-globin* gene was detectable in all the swab samples (100%), then we defined those samples with *β-globin* level below 10^5^ copies/swab as unqualified ones, and thus excluded them from further analysis. Among the remaining 905 samples, the mean of *β-globin* gene was 2.6×10^6^ copies/swab, median 2.6×10^6^ copies/swab, and range from 1.2×10^5^ to 1.2×10^8^. EBV DNA from these nasopharyngeal swabs was positively detected (>0 copies/swab) in 89% (802/905) of the samples, while 11% (103/905) were undetectable (0 copies/swab). The EBV load distribution of the 802 subjects with detectable EBV was unimodal, with a mean of 6.0×10^3^ copies/swab (median 6.7×10^3^, range from 6 to 1.1×10^8^). At the 1^st^-round follow-up retest, 94% (847/905) of subjects maintained VCA/IgA positive status (≥1:5), with a mean IgA antibody titer 1:10.7 (median 1:10, range 1:5–1:160), while 6% (48/905) of individuals VCA/IgA turned negative. The mean EBV load from VCA/IgA positive subjects was higher than that of VCA/IgA negatives (Z = -1.976, *P* = 0.048, Mann-Whitney U test). Reciprocally, mean VCA/IgA titer among EBV DNA positive subjects was higher than that of EBV negatives (Z = -2.870, *P* = 0.004, Mann-Whitney U test) (Table 2).

**Table 2:** Nasopharyngeal EBV DNA load and serum VCA/IgA titers in seropositive high-risk population.

Parameter		Numbers	Log10(EBV DNA copies)			Log10(serum VCA/IgA titers)		
			Mean	SD	Range	Mean	SD	Range
**VCA/IgA**								
(-)		58	3.01	1.60	0–6.58			
(+)		847	3.38	1.52	0–8.03	1.03	0.25	0.70–2.20
1:5		201	3.06	1.46	0–5.78	0.70	0.00	0.70–0.70
1:10		415	3.26	1.52	0–6.53	1.00	0.00	1.00–1.00
1:20		197	3.75	1.40	0–7.45	1.30	0.00	1.30–1.30
1:40		21	4.42	1.46	0–8.03	1.60	0.00	1.60–1.60
≥1:80		13	4.47	1.60	0–6.53	1.93	0.00	1.90–2.20
**EBV DNA**								
(-)		103				0.87	0.36	0–1.90
(+)		802	3.78	0.99	0.78–8.03	0.97	0.35	0–2.20
**EBV DNA**	**VCA/IgA**							
(-)	(-)	10						
(-)	(+)	93				0.97	0.22	0.70–1.90
(+)	(-)	48	3.64	0.88	2.15–6.58			
(+)	(+)	754	3.79	1.00	0.78–8.03	1.04	0.26	0.70–2.20

EBV, Epstein-Barr virus; VCA, viral capsid antigen; SD, standard deviation; (+), positive; (-), negative.

**Fig 1:**
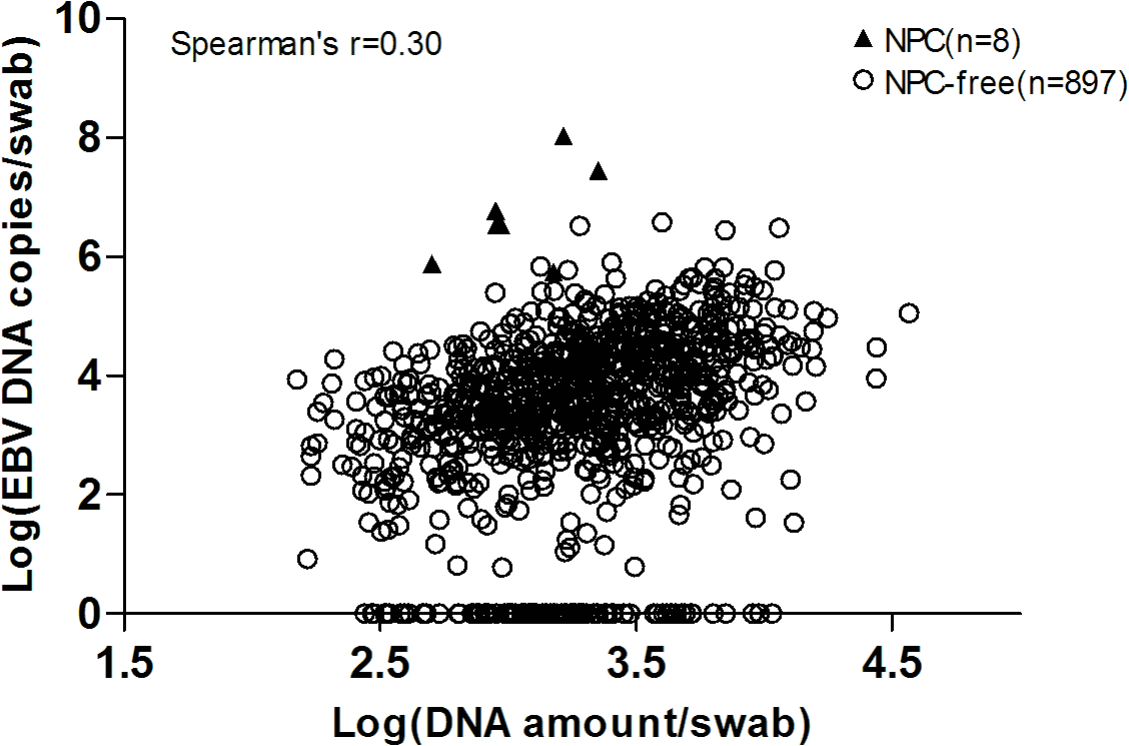
Relationship of EBV DNA load and DNA amount in the nasopharyngeal swabs. The swab EBV DNA load showed only a modest correlation with the DNA amount in the nasopharyngeal swabs (Spearman’s correlation coefficient = 0.30, *P* < 0.001).

To examine whether swab sampling variation influenced EBV load, Spearman’s correlation coefficient between copy number of EBV DNA and total DNA amount per swab was calculated, and the result showed only a modest correlation (Spearman’s correlation coefficient = 0.30, *P* < 0.001) (Fig 1). This indicated that swab sampling variation was not a major determinant of EBV load. This observation was further corroborated by the similar results irrespective of whether we used EBV load per swab or EBV load normalizing with *β-globin* in the analyses. Thus in the main text we presented only results based on the copy number of EBV DNA per swab.

### Viral load and its correlation with serum anti-EBV VCA/IgA titers

Among 905 subjects, 754 were positive for both EBV DNA and VCA/IgA, and 10 negative for the 2 tests, with an overall concordance rate of 84.4%. And the *PABAK* coefficient was 0.688, showing high agreement of the 2 tests when being treated as independent variable. For the 48 EBV positive but VCA negative subjects, the EBV load was not statistically different from the dual positive group (Z = -1.455, *P* = 0.146, Mann-Whitney U test; Table 2), while the figure for the 93 EBV negative and VCA positive group, the VCA/IgA level showed a statistical difference in comparison with the dual positive group (Z = -2.521, *P* = 0.012, Mann-Whitney U test; Table 2). Notwithstanding, when we took into account quantitative results of the 2 tests, we found that nasopharyngeal EBV copy numbers increased with increasing VCA/IgA antibody titers, with a weak correlation observed (Spearman’s correlation coefficient = 0.229, *P* < 0.001; Table 2). The result remained unchanged after multivariate adjustment for age, sex, and swab *β-globin* copy numbers (Table 3).

**Table 3:** Multivariate liner regression for the relation between age, sex, β-globin copy numbers, serum VCA/IgA and nasopharyngeal EBV DNA load.

Variables	Dependent [Y = log10(EBV DNA copies)]		
	beta	t	*P* value
Age	0.02	3.85	<0.001
Sex	0.22	2.20	0.028
Log10(*β-globin* copies)	0.62	6.92	<0.001
VCA/IgA(1:5)^a^	-0.02	-0.07	0.942
VCA/IgA(1:10)^a^	0.16	0.81	0.418
VCA/IgA(1:20)^a^	0.61	2.82	0.005
VCA/IgA(1:40)^a^	1.21	3.29	0.001
VCA/IgA(≥1:80)^a^	1.73	3.93	<0.001

EBV, Epstein-Barr virus; VCA, viral capsid antigen.

^a^ Compared to VCA/IgA negative group (reference).

### Viral load and VCA/IgA titers by age and sex

**Fig 2:**
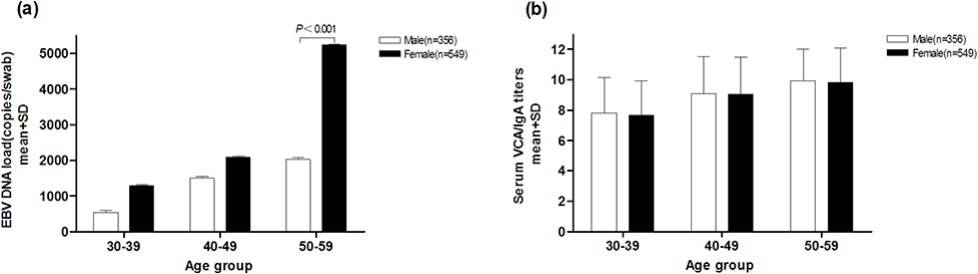
EBV load and VCA/IgA titers in males and females. Nasopharyngeal EBV load and serum VCA/IgA titers by gender and age groups. (a) Mean EBV load in females was higher than that of males by different age groups; EBV load increased with age in both genders. (b) There was no difference in VCA/IgA titers between males and females in different age groups; VCA/IgA titers increased with age in both males and females.

In the high-risk subjects, 356 were males and 549 were females, and the mean age was 49.1±8.3 and 47.4±9.3, respectively. We found that the swab EBV load in females was higher than that in males with a ratio of nearly 2 fold (mean: 2.9×10^3^ vs 1.5×10^3^ copies/swab; Z = -3.257, *P* = 0.001, Mann-Whitney U test; Table 4). Whereas there was no statistically significant difference between males and females for VCA/IgA titers (mean, 1:9.3 vs 1:9.1; Z = -0.372, *P* = 0.710, Mann-Whitney U test; Table 4). A weak correlation was observed between EBV load and age (Spearman’s correlation coefficient = 0.139, *P* < 0.001; Table 4). The figure was similar for VCA/IgA with a Spearman’s correlation coefficient = 0.104 (*P* = 0.002; Table 4). A higher EBV load level was still found in females when dividing into age groups, although statistically significant difference was only present in 50~59 age group (Z = -4.372, *P* < 0.001, Mann-Whitney U test; Fig 2A). And the EBV load and serology showed an increased trend along with age both in males and females when the analyses were stratified by sex. (Fig 2A and 2B).

**Table 4:** Nasopharyngeal EBV DNA load and serum VCA/IgA titers by gender and age.

Parameter	Numbers	Log10(EBV DNA copies)				Log10(serum VCA/IgA titers)			
		Mean	SD	Range	*P* value^*^	Mean	SD	Range	*P* value^*^
**Gender**									
Male	356	3.18	1.63	0–8.03	0.001	0.97	0.35	0–2.20	0.710
Female	549	3.46	1.44	0–6.58		0.96	0.35	0–1.90	
**Age group**									
30–34	86	2.99	1.61	0–5.75		0.91	0.36	0–1.60	
35–39	95	3.02	1.52	0–6.77		0.87	0.36	0–1.30	
40–44	156	3.22	1.55	0–6.58		0.94	0.39	0–2.20	
45–49	113	3.30	1.68	0–7.45		0.97	0.31	0–1.90	
50–54	161	3.51	1.48	0–6.45		0.98	0.35	0–1.90	
55–59	294	3.57	1.41	0–8.03		1.00	0.33	0–1.90	
Total	905	3.35	1.52	0–8.03		0.96	0.35	0–2.20	

EBV, Epstein-Barr virus; VCA, viral capsid antigen; SD, standard deviation.

^*^ Mann-Whitney U test, α = 0.05.

### Comparison of viral load and VCA/IgA titers between NPC and NPC-free seropositive high-risk subjects

**Fig 3:**
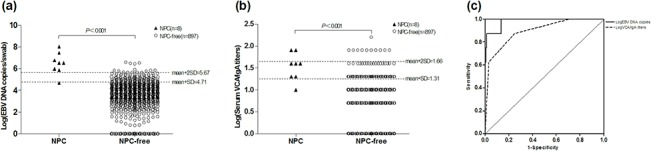
Diagnostic performance of EBV load and VCA/IgA titers. Cut-off values (COV) and areas under receiver operating characteristic (ROC) curves were calculated to evaluate the diagnostic performance of EBV load and VCA/IgA titers. (a) The optimal COV for EBV load was mean plus 2 standard deviations (i.e. 4.7×10^5^ copies/swab); (b) The best COV for VCA/IgA titers was mean plus standard deviation (i.e. 1:20); (c) The ROC curve indicated that EBV load had a better diagnostic value than VCA/IgA titers; the area under the curve of EBV load was larger than VCA/IgA titers.

During follow-up, 8 individuals were diagnosed with NPC by pathological examination in the 905 NPC high-risk subjects (Table 5). Seven of the 8 cases were early-stage NPCs, and the early diagnosis rate was 87.5%. All the 8 NPC cases were undifferentiated and non-keratinizing carcinoma. Six of them were males and two were females, and the cumulative incidence rates among males was 1.69% (6/356), and among females 0.36% (2/549) (male vs female, RR = 4.69, 95%CI: 0.94–23.36). The mean EBV load among the NPCs was significantly higher than that of NPC-free high-risk individuals (mean, 2.8×10^6^ copies/swab [range 4.8×10^4^−1.1×10^8^] vs 5.6×10^3^ copies/swab [range 0–3.8×10^6^]; Z = -4.688, *P* < 0.001, Mann-Whitney U test) (Fig 3A). The mean titer of VCA/IgA was also significantly higher in NPCs than that of NPC-free subjects (NPC vs NPC-free = 1:33.9 vs 1:9.1; Z = -4.097, *P* < 0.001, Mann-Whitney U test) (Fig 3B). Both the EBV load and VCA/IgA titer of the NPC patients showed no correlation with the clinical stage (Spearman’s correlation coefficient = 0.385, *P* = 0.346 and 0.193, *P* = 0.648, respectively).

**Table 5:** Comparison of viral load and VCA/IgA titers between NPC and NPC-free high-risk subjects.

Parameter	Numbers	Log10(EBV DNA copies)			Log10(serum VCA/IgA titers)		
		Mean	SD	Range	Mean	SD	Range
**NPC cases** ^a^	8	6.45	1.04	4.68–8.03	1.53	0.31	1.00–1.90
StageⅠ	2	6.26	0.72	5.75–6.77	1.45	0.21	1.30–1.60
StageⅡ	5	6.21	1.03	4.68–7.45	1.54	0.39	1.00–1.90
StageⅢ	1	8.03			1.60		
Male	6	6.84	0.80	5.75–8.03	1.45	0.32	1.00–1.90
Female	2	5.28	0.84	4.68–5.88	1.75	0.21	1.60–1.90
**NPC-free subjects**	897	3.75	0.96	0–6.58	0.96	0.35	0.00–2.20
Male	350	3.12	1.57	0–6.52	0.96	0.35	0.00–2.20
Female	547	3.46	1.44	0–6.58	0.95	0.35	0.00–1.90
**Comparision:**							
NPC vs NPC-free for EBV load ^*^		Z = -4.688, *P*<0.001					
NPC vs NPC-free for VCA/IgA ^*^					Z = -4.097, *P*<0.001		

EBV, Epstein-Barr virus; VCA, viral capsid antigen; SD, standard deviation; NPC, nasopharyngeal carcinoma.

^a^ All the 8 NPC cases were undifferentiated and non-keratinizing carcinoma

^*^ Mann-Whitney U test, α = 0.05.

### Comparison of diagnostic performance between VCA/IgA titers and EBV load

Series of cut-off values (COV) of VCA/IgA titers and nasopharyngeal EBV load were defined by calculating mean plus standard deviation (SD) from NPC-free population (Table 6). The COV of EBV load was tried as 3 different levels: mean+SD, mean+2SD, mean+3SD, and the COV as mean+2SD (i.e. 4.7×10^5^ copies/swab; Table 6, Fig 3A) showed an optimal diagnostic performance, with sensitivity, specificity, positive predictive value, negative prediction value being 87.5%, 98.9%, 41.2%, and 99.9%, respectively. More than 95% subjects in this study had a value lower than the COV, meaning that over 95% of high risk individuals could be excluded from further follow-up. In contrast, the best COV of VCA/IgA was mean+SD (i.e. 1:20; Table 6, Fig 3B), and the corresponding sensitivity, specificity, positive predictive value, negative prediction value were 87.5%, 75.0%, 3.1%, and 99.9%, respectively. Receiver operating characteristic curves (ROC curve) were created to evaluate the overall diagnostic value of these two markers (Fig 3C); the area under the curve (AUC) of EBV load was larger than that of VCA/IgA (0.980 vs 0.895) (Table 7). Nasopharyngeal EBV load therefore showed a better diagnostic value than VCA/IgA in NPC mass screening among high-risk individuals.

**Table 6:** Cut-off values for EBV load and VCA/IgA titers.

Parameter	Cut-off value for EBV load (copies/swab)						Cut-off value for VCA/IgA			
	COV = Mean+SD		COV = Mean+2SD		COV = Mean+3SD		COV = Mean+SD		COV = Mean+2SD	
	(5.1×10^4^)		(4.7×10^5^)		(4.3×10^6^)		(1:20)		(1:45)	
	Above	Below	Above	Below	Above	Below	Above	Below	Above	Below
NPC(n = 8)	7	1	7	1	3	5	7	1	2	6
NPC-free(897)	121	776	10	887	0	897	224	673	11	886
Sensitivity	87.5%		87.5%		37.5%		87.5%		25.0%	
Specificity	86.5%		98.9%		100.0%		75.0%		98.8%	
PPV	5.5%		41.2%		100.0%		3.0%		15.4%	
NPV	99.9%		99.9%		99.4%		99.9%		99.3%	

EBV, Epstein-Barr virus; VCA, viral capsid antigen; COV, cut-off value; SD, standard deviation; NPC, nasopharyngeal carcinoma; PPV, positive predictive value; NPV, negative prediction value.

**Table 7:** Area under the operating characteristics curve.

Parameter	Area	*P* value	95% Confidence Interval
Log10(EBV DNA Copies)	0.980	<0.001	0.949–1.012
Log10(VCA/IgA titers)	0.895	<0.001	0.782–1.007

## Discussion

To our knowledge, this study is the first prospective study that investigated the EBV DNA load in the nasopharynx of seropositive high-risk individuals. The results indicated that nasopharyngeal EBV copy numbers can be a useful tool to predict NPC occurrence among serologically defined high-risk individuals.

EBV is carried by more than 90% of the adult population worldwide as a largely nonpathogenic infection [20, 21]. Primary infection is usually asymptomatic, and the virus subsequently persists lifelong in memory B cells in the peripheral blood of infected individuals as latent state, with a restriction of viral gene expression or even completely absent thereby allowing EBV to remain hidden from the immune system. In most of the cases, the virus is replicated and infectious virions can be recovered in saliva. This replication that results in the release of infectious virus is referred to as the EBV lytic phase of the viral life cycle. It is assumed that activation of the lytic phase occurs in memory B cells differentiating into plasma cells when recirculating through the lymphoid tissue associated with the oropharyngeal mucosa [22]; The activated EBV in mucosal epithelium may lead to the increase of serum IgA antibodies such as VCA/IgA. Even though EBV infection persists during the hosts' life, only a very small proportion of the hosts maintaining elevated IgA titers to EBV are defined as NPC high risk population. However, the mechanism underlying viral reactivation in vivo, especially in the nasopharynx is not clearly understood.

Although the correlation was modest, our results showed that serum VCA/IgA antibody titers increased with increasing nasopharyngeal EBV load, indicating that the EBV reactivation in the nasopharynx is directly responsible for elevated serum EBV IgA antibody titers. Interestingly, as known, the incidence of NPC is 2 to 3 fold higher in males than in females, but the present study found that the nasopharyngeal EBV load in females was higher than males. This might be due to the different hormone levels and genetic background of males and females, which, as a result, lead to distinct nasopharyngeal microenvironments. Further and more investigations should be conducted to explain this phenomenon.

Even by combining tests of several EBV-related antibodies, the effectiveness of NPC screening remains relatively low. In a population based study in Zhongshan city, only 4.4% of high-risk populations developed NPC within the first year of follow-up, while nearly 95% of healthy individuals nevertheless received more invasive and expensive additional diagnostic tests, such as nasopharyngeal endoscopy and biopsy examinations [9]. In the present study, for the high-risk individuals, the approach of an additional nasopharyngeal swabbing allowed us to predict the presence of lesions in the nasopharynx, since almost 100% of NPC cells have EBV DNA and nasopharyngeal epithelium of premalignant lesions harbors clonal EBV [2, 13]. Individuals with absence of EBV DNA in the nasopharyngeal swab might have ignorable possibility to develop NPC. Our current study found that 11% of high-risk individuals had 0 copies in the nasopharyngeal swabs. After three years of follow-up, none of them developed NPC. So these individuals should be ruled out for further diagnostic evaluation. If we take a further step by calculating mean plus two SD from NPC-free population as cut-off value of nasopharyngeal EBV load, i.e. 4.7×10^5^ copies/swab, the diagnostic performance was improved significantly, with sensitivity, specificity, positive predictive value, negative prediction value 87.5%, 98.9%, 41.2%, and 99.9%, respectively. More importantly, over 95% subjects in this study were under the cut-off value and could be excluded from further follow-up. While for VCA/IgA, the best cut-off value was mean plus SD (i.e. 1:20), and the sensitivity, specificity, positive predictive value, negative prediction value were 87.5%, 75.0%, 3.1%, and 99.9%, respectively. The screening efficiency was low and most of the subjects exceeded the cut-off value and needed to undergo close follow-up. In this regard, using 4.7×10^5^ copies of nasopharyngeal EBV load as cut-off value, our strategy of an additional test of EBV load in the nasopharynx might narrow down the number of unnecessary invasive diagnostic examinations, thus improving cost-effectiveness and subject compliance in NPC screening programs. A case-control study carried out in Indonesia in which diagnostic value of EBV load in nasopharyngeal brush was estimated also showed that diagnostic performance of nasopharyngeal EBV load was superior to EBV IgA serological test [23].

Current gold standard of clinical NPC detection is nasopharyngeal endoscopy combined with biopsy of suspicious lesions, while early diagnosis of NPC is sometimes difficult with endoscopy. Endoscopy presentations may be subtle or obscure in early NPC lesions, e.g., only a slight fullness in the Rosenmüller’s fossa, or a small bulge in the roof. Clinically, 13.8% of NPCs spread submucosally, and endoscopic detection misses as high as 51.4% of NPC patients with submucosal growth pattern [24]. To make definite NPC diagnosis and avoid misdiagnosis of subclinical cancers, it is not uncommon to perform multiple biopsies for some patients. Additional MRI scanning may be helpful to depict subclinical cancers missed at endoscopy [25]. Several techniques such as narrow-band imaging, contact endoscopy, and very recently, trans-oral brush biopsies have been developed to enhance the diagnostic sensitivity of endoscopy [26–28]. However these techniques are either invasive or expensive, and most importantly, cannot be performed by general physicians, thus, they are not suitable for mass screening programs, especially in the rural areas where only limited health care facilities are available. In contrast, our current and previous studies demonstrated that nasopharyngeal swabbing is non-invasive, sensitive, simple, low-cost and easy to perform by non-otorhinolaryngology physicians or paramedical personnel. These attributes together make our method extraordinary amenable for the field study of large-scale population-based NPC screening programs.

In conclusion, in this prospective and population-based study we demonstrated that an additional assay of EBV DNA load in the nasopharynx among high-risk individuals may reduce the number of subjects needed to be closely followed up. Future studies using other serological assays (e.g. ELISA), and using a non-repetitive segment of the EBV genome for a more precise quantification of the EBV DNA or with larger sample sizes and longer follow-up duration are warranted to verify our results. If confirmed, quantification of nasopharyngeal EBV DNA load among subjects with elevated EBV antibody levels should be added as supplementary tool in the NPC screening program in high-risk populations.
